# The styrene–maleic acid copolymer: a versatile tool in membrane research

**DOI:** 10.1007/s00249-015-1093-y

**Published:** 2015-12-06

**Authors:** Jonas M. Dörr, Stefan Scheidelaar, Martijn C. Koorengevel, Juan J. Dominguez, Marre Schäfer, Cornelis A. van Walree, J. Antoinette Killian

**Affiliations:** Membrane Biochemistry and Biophysics, Bijvoet Center for Biomolecular Research and Institute of Biomembranes, Department of Chemistry, Faculty of Science, Utrecht University, Padualaan 8, 3584 CH Utrecht, The Netherlands; School of Chemical and Physical Sciences, Flinders University, GPO Box 2100, Adelaide, 5001 Australia

**Keywords:** Membrane proteins, Native nanodiscs, Styrene–maleic acid copolymers, SMALPs, Lipid–protein interactions, Lipodisq

## Abstract

A new and promising tool in membrane research is the detergent-free solubilization of membrane proteins by styrene–maleic acid copolymers (SMAs). These amphipathic molecules are able to solubilize lipid bilayers in the form of nanodiscs that are bounded by the polymer. Thus, membrane proteins can be directly extracted from cells in a water-soluble form while conserving a patch of native membrane around them. In this review article, we briefly discuss current methods of membrane protein solubilization and stabilization. We then zoom in on SMAs, describe their physico-chemical properties, and discuss their membrane-solubilizing effect. This is followed by an overview of studies in which SMA has been used to isolate and investigate membrane proteins. Finally, potential future applications of the methodology are discussed for structural and functional studies on membrane proteins in a near-native environment and for characterizing protein–lipid and protein–protein interactions.

## Introduction

The study of integral membrane proteins (MPs) is one of the major challenges in current research in molecular life sciences. MPs represent a substantial fraction of protein-encoding genes (Wallin and von Heijne 1998), they fulfill a variety of essential functions in all organisms (von Heijne 2007), and they have high pharmacological relevance (Overington et al. 2006). Despite the evident importance of these proteins, our understanding of the principles that govern folding, stability, and function of MPs remains poor as compared to water-soluble proteins. Indeed, structures of MPs are largely underrepresented in the protein database: to date only 556 unique structures of MPs have been deposited (White 2015), accounting for less than 2 % of all structures. This discrepancy is not due to a lower biological abundance or relevance of MPs, but is mainly caused by difficulties in experimental approaches to study these hydrophobic proteins. Under native conditions, MPs are embedded into biomembranes, an anisotropic environment established by a bilayer of amphipathic lipids with a hydrophobic core that shields the hydrophobic surface of the proteins from the aqueous phase. For detailed structural and functional studies however, MPs need to be isolated from this complex environment and purified while maintaining both their stability and activity. This has proven to be a far more demanding task than the isolation and purification of soluble proteins and thus much effort has been focused on new methodologies for improved MP solubilization and stabilization. A promising new approach is the use of styrene–maleic acid (SMA) copolymers to solubilize MPs directly in their native environment in the form of polymer-bounded nanodiscs.

In this article, we will first briefly review the state of the art in membrane protein solubilization and stabilization, including an introduction to the SMA method. We will continue with a description of the properties of SMA copolymers and then discuss studies in which model membrane systems are used to investigate the mode of action of SMA and to characterize the nanodiscs. We then will illustrate the potential of the methodology by presenting an overview of recent studies in which SMA has been successfully used to isolate and investigate a wide range of MPs from different biological sources. In the last section we will discuss potential future applications of the use of SMA, in particular with respect to studying structural and functional properties of MPs and characterizing interactions between membrane components.

## Membrane protein solubilization and stabilization

**Fig. 1:**
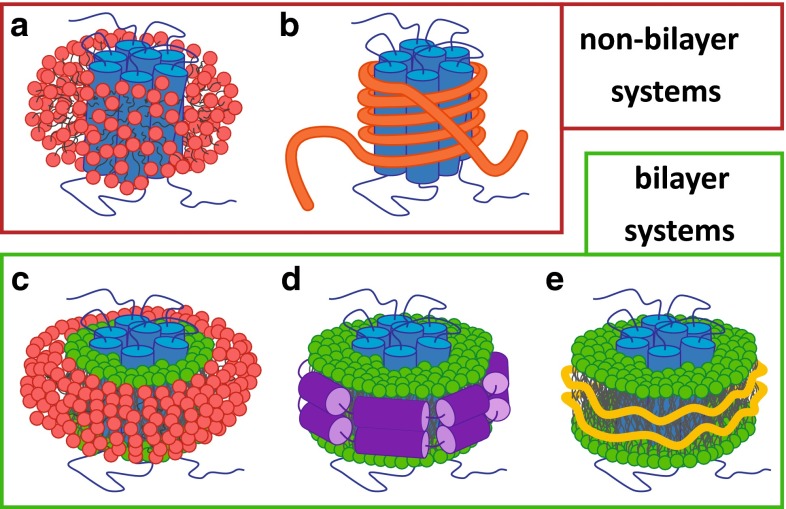
Membrane-mimetic systems for membrane protein stabilization. The protein is indicated in *blue* and lipids in bilayers are indicated in *green*. **a** Protein in detergent (*red*) micelle. **b** Protein stabilized by amphipol (*orange*). **c** Protein in bicelle (detergent in *red*). **d** Protein in nanodisc stabilized by MSP (*purple*). **e** Protein in nanodisc stabilized by SMA (*yellow*)

One of the largest challenges in membrane protein solubilization lies in finding an environment with optimal properties to allow a variety of downstream studies. Ideally, this environment should stabilize the protein, allow for its purification, and enable the study of its structural and functional properties while the protein displays native behavior. Figure 1 illustrates some of the membrane-mimetic systems that are commonly used in membrane protein research. The various approaches include the use of detergents for solubilization into micelles (Fig. 1a) and replacement of detergent by more stabilizing agents, such as amphipols (Fig. 1b). In addition, MPs can be reconstituted into a lipid bilayer-forming environment such as bicelles (Fig. 1c), lipid vesicles (not shown), or nanodiscs that are stabilized by membrane scaffold proteins (MSPs) (Fig. 1d). A recently developed alternative approach is the use of SMA copolymers to directly solubilize membranes in the form of nanodiscs (Fig. 1e). In this section, we will give a brief overview of these different approaches and discuss some of their advantages and disadvantages.

### Detergents

A common strategy for MP isolation is the solubilization of the lipid bilayer matrix with detergents (Garavito and Ferguson-Miller 2001), which generally leads to the formation of spherical micelles, comprising MPs, detergent molecules, and possibly some remaining lipids (le Maire et al. 2000; Lichtenberg et al. 2013). To achieve extraction of different MPs from membranes with varying properties, a wide range of detergents with high solubilizing efficiency has been utilized (for reviews see Moraes et al. 2014; Privé 2007; Seddon et al. 2004). Although this approach without any doubt has contributed much to our understanding of MPs, detergent solubilization has some inherent disadvantages. First, working with an MP with unknown properties requires an extensive, mainly empirical screening to find a suitable detergent (mix) for each specific case (Arachea et al. 2012; Privé 2007). Second, detergent addition strips the protein of its native lipid environment and thus generally leads to a loss of native interactions with both lipids and other proteins. Third, and probably most importantly, detergent micelles are a rather poor mimic of a lipid bilayer since they exhibit very different physico-chemical properties (Bordag and Keller 2010; Zhou and Cross 2013a). Micelles have a single hydrophilic surface that is highly curved and their hydrophobic parts show a low degree of order. Furthermore, detergent molecules are subject to a monomer–micelle equilibrium causing monomers to rapidly exchange between the micellar and soluble pool, thereby unfavorably increasing the dynamics of the protein environment. In addition, water permeability and lateral pressure profiles differ extensively in micelles and bilayers. As a result, MPs generally show a lower stability in micelles and transient solvent exposure of the hydrophobic MP surface can lead to inactivation or aggregation of the protein. Furthermore, the use of detergents may interfere with MP function (Quick et al. 2012) or cause the protein to adopt a non-physiological conformation (Zhou and Cross 2013b; Zoonens et al. 2013). Because of these problems, much effort is being directed towards the development of new detergents with improved properties. In particular, fluorinated compounds (Frotscher et al. 2015) and maltose neopentyl glycol detergents (Chae et al. 2010) have proven to be powerful with respect to their ability to stabilize MPs and hence may develop into more general tools in membrane research.

### Amphipols

An alternative option to achieve a more stabilizing environment for MPs is the replacement of detergent by other classes of specially designed molecules. Promising approaches include facial amphiphiles (Zhang et al. 2007) and a variety of peptide surfactants (Koutsopoulos et al. 2012 and references therein; Tao et al. 2013), but perhaps the most notable and generally applicable approach at this moment is the use of amphipols. This class of amphipathic polymers, developed by Popot and coworkers, comprises a polyacrylate backbone that is equipped with pendant hydrophobic and hydrophilic sidechains (Tribet et al. 1996). Amphipols provide a versatile platform for investigations of MPs since they significantly improve the stability of MPs in general and since MP–amphipol complexes are amenable to a plethora of biophysical studies (for reviews see Popot et al. 2011; Zoonens and Popot 2014). These complexes are characterized by low exchange rates of protein-bound amphipols and free monomers in solution, which results in a high stability and allows the use of relatively low concentrations of free amphipols as compared to detergent. A further advantage of the use of amphipols is that the variety of sidechains with different functional groups allows for optimization of these polymers for specific applications as well as for chemical modification and introduction of labels such as fluorophores.

### Vesicles and bicelles

A drawback of the membrane-mimetic systems discussed above is the lack of an actual lipid bilayer environment. Such an environment is important because its particular physico-chemical properties may be essential for structure, function and stability of MPs (Zhou and Cross 2013a). One way to overcome this problem is the reconstitution of MPs into systems of synthetic lipids such as planar lipid bilayers or lipid vesicles (Kiessling et al. 2008; Oiki 2015; Rigaud and Lévy 2003). These systems allow compartmentalization and hence MP-mediated vectorial transport can be studied. In addition, they enable systematic investigation of the effect of the membrane lipid composition on structural and functional properties of MPs. However, these systems also have limitations: planar and supported bilayers for example are immobilized systems and are thus not suitable for solution-based methods, whereas vesicles have a relatively large size that may impede optical spectroscopy due to light scattering.

An alternative is the use of bicelles, which are discoidal structures obtained by mixing phospholipids with detergents (often short-chain phospholipids) in a defined ratio (for a review see Dürr et al. 2013). Depending on their composition, bicelles can have different sizes ranging from 8–50 nm in diameter (Vold and Prosser 1996). The larger specimens are particularly useful for nuclear magnetic resonance (NMR) spectroscopy since they can orient in the magnetic field (Howard and Opella 1996). However, bicelles are limited to certain lipid compositions and stability is often a problem.

### Nanodiscs bounded by membrane scaffold proteins

A relatively new approach to incorporate MPs in a lipid bilayer environment was developed by Sligar and coworkers. They designed a method to transfer MPs from detergent micelles into lipid nanodiscs, which are small patches of a lipid bilayer, bounded by membrane scaffold proteins (MSPs) (Bayburt et al. 2002). To achieve this, they engineered amphipathic helical proteins derived from human apolipoprotein A-1 that serve to shield the hydrophobic core of the lipids from the aqueous phase. Reconstitution of MPs into these soluble particles seems to be generically applicable irrespective of the type of protein and they convey a relatively high protein stability (for reviews see Bayburt and Sligar 2010; Schuler et al. 2012). The diameter of nanodiscs is typically in the order of ~10 nm, but generation of specific MSP variants allows the formation of smaller (~6–7 nm) (Hagn et al. 2013; Wang et al. 2015) and larger (16–17 nm) (Grinkova et al. 2010) nanodiscs. Furthermore, the use of different apolipoproteins or derived peptides and the variation of the peptide/protein–lipid ratio enable the preparation of larger particles (Chromy et al. 2007; Park et al. 2011). This control over size renders them excellent tools in many biophysical methods for structural and functional characterization of MPs. In addition, MSPs can be modified by genetic engineering, which allows for functionalization and for the introduction of labels or affinity tags. Like in other bilayer systems, the lipid composition in nanodiscs can be controlled, enabling systematic studies (Bayburt et al. 2010). MPs can even be incorporated into nanodiscs with exclusively native lipid material from detergent-solubilized membranes (Civjan et al. 2003). An additional advantage of nanodiscs is that, in contrast to other bilayer systems, MPs can be trapped in a defined oligomeric state, allowing studies on how oligomerization influences protein function (Boldog et al. 2006; Shi et al. 2012). MSP nanodiscs thus are a particularly promising system for MP research and they are being used in a growing number of studies.

### Nanodiscs bounded by styrene–maleic acid copolymers

It is clear that much progress has been made in optimizing different environments to stabilize MPs. However, all systems described above have one common disadvantage: they all require detergents to extract native MPs from cellular membranes. Therefore, the problem of transient protein destabilization by detergent persists for MP reconstitution into both membrane-mimetic and bilayer systems. In order to attenuate this problem, alternative approaches are being developed, such as cell-free protein production (Roos et al. 2012; He et al. 2015), MP-enriched cell-derived extracelluar vesicles (Zeev-Ben-Mordehai et al. 2014), genetic engineering of the MP by fusion (Mizrachi et al. 2015) or minimization of the exposure time with detergent (Shirzad-Wasei et al. 2015). However, arguably the most promising new method as an alternative to detergent extraction has become available by the recent discovery of the membrane-solubilizing effect of amphipathic SMA copolymers (Knowles et al. 2009; Tighe and Tonge 2000; Tonge 2006), as will be discussed in detail in the next sections.

SMA molecules exhibit a distinctly different mode of action than detergents: addition of the polymer to synthetic or biological lipid membranes leads to the spontaneous formation of discoidal particles with diameters of ~10 nm. In this new type of polymer-bounded nanodiscs, the bilayer organization of the incorporated lipid molecules is conserved (Jamshad et al. 2015b; Orwick et al. 2012). In different studies, these particles have been referred to as SMA–lipid particles (SMALPs) (Knowles et al. 2009), Lipodisq particles (Orwick et al. 2012), or native nanodiscs (Dörr et al. 2014). Depending on the origin of the lipid material, we here use the terms *SMALPs* for particles derived from synthetic liposomes and *native nanodiscs* for isolations from biological membranes.

The most striking feature of this novel system is the possibility to directly extract MPs from cells without an intermediate step of conventional detergent solubilization (Long et al. 2013). Thus, the native nanodisc system combines a solubilizing power similar to detergents with the small particle size of nanodiscs, while conserving a minimally perturbed native lipid environment that stabilizes the protein. To date, this method has been used in a number of reports employing various biochemical and biophysical techniques to study MPs, as will be discussed in more detail later.

## The styrene–maleic acid copolymer

### Copolymers of styrene and maleic acid/anhydride: chemical structure, applications, and availability

**Fig. 2:**
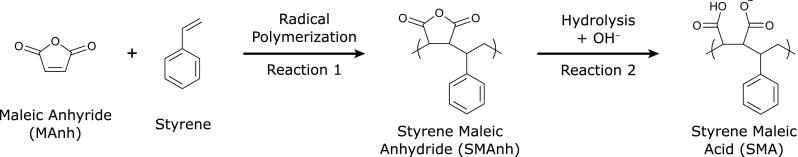
Schematic representation of the synthesis of styrene–maleic anhydride (Reaction 1) and the preparation of styrene–maleic acid (Reaction 2) as illustrated here for a 1:1 styrene-to-maleic anhydride/acid molar ratio. When styrene is present in excess, the monomer sequence distribution in the polymer becomes more complex (see text for details)

Styrene–maleic acid (SMA) is the hydrolyzed form of the styrene–maleic anhydride (SMAnh) copolymer, which is synthesized by the copolymerization of styrene and maleic anhydride monomers (Fig. 2, Reaction 1). Both forms of the polymer are widely used in industry and they have many different applications. For instance, SMAnh is commonly used as thermal stabilizer in plastic blends, while SMA can be used as a dispersing agent for ink formulations and coatings. The SMA/SMAnh copolymers are produced by several suppliers worldwide. The major ones are TOTAL Cray Valley (Beaufort, TX, USA) and Polyscope (Geleen, NL), the latter using the brand name “Xiran” for their SMA/SMAnh copolymers. The products are typically sold in large quantities to companies that process the polymers for downstream products.

SMA copolymers also have a long-standing history in life sciences, originally being described as conjugates for drugs in cancer therapy (Maeda et al. 1979; Maeda 2001). Later, it was found that SMA can interact with phospholipids to form discoidal structures that can incorporate hydrophobic molecules and therefore would be useful as a drug delivery system (Tighe and Tonge 2000; Tonge and Tighe 2001). Based on this observation, new applications using SMA for the solubilization of lipid bilayers were developed and commercialized, as described in a patent by Malvern Cosmeceutics (Worcester, UK) (Tonge 2006). In particular, the application of SMA to solubilize membrane proteins, as first reported by the groups of Dafforn and Overduin (Knowles et al. 2009), has led to a rapidly increasing interest in SMA as a novel tool in membrane research. Following these developments, SMA/SMAnh copolymers are now also commercially available in small quantities from Sigma Aldrich (St. Louis, MO, USA).

Both SMA and SMAnh copolymers can be obtained in different commercial grades that vary in styrene–maleic anhydride/acid ratio and in average molecular weight. However, even within a single preparation of SMA/SMAnh copolymers there are large variations in molecular weight and in composition. The reason for this lies in the synthesis of SMAnh, as will be discussed next.

### Synthesis and composition of styrene–maleic anhydride copolymers

The polymerization of styrene and maleic anhydride (MAnh) monomers (Fig. 2, Reaction 1) is a radical chain reaction that leads to the formation of SMAnh copolymers with a wide distribution in molecular weights. This distribution is characterized by the so-called polydispersity index (PDI), which for SMA is typically in the range of 2.0–2.5. The PDI is defined as the ratio of the weight-average molecular weight (*M*_w_) and the number-average molecular weight (*M*_n_) as:

$${\text{PDI }} \equiv \frac{{M_{\text{w}} }}{{M_{\text{n}} }};\quad M_{\text{w}} \equiv \frac{{\mathop \sum \nolimits M_{i}^{2} n_{i} }}{{\mathop \sum \nolimits M_{i} n_{i} }};\quad M_{\text{n}} \equiv \frac{{\mathop \sum \nolimits M_{i} n_{i} }}{{\mathop \sum \nolimits n_{i} }} ,$$

where *n*_*i*_ is the number of polymer molecules of a molecular weight *M*_*i*_. The concept of PDI can be illustrated by a simple calculation. Let us consider a distribution of three polymer molecules with molecular weights of 500, 1000, and 10,000 Da. In this example, *M*_w_ is ~8800 Da, and *M*_n_ is ~3900 Da, resulting in a PDI of ~2.25. For a typical SMAnh polymer with a PDI of ~2.5 this means that the polymer chains have a broad size distribution with the smallest and largest chains differing by more than at least one order of magnitude in molecular weight and thus chain length.

The styrene–maleic anhydride/acid monomer ratio in the polymer can simply be varied by changing the feed monomer ratio used in the polymerization process. However, the resulting polymer generally does not consist of regular repeating building blocks of styrene and MAnh units with this feed ratio, nor does it exhibit a completely random distribution of the monomers along the chain. This is because of the differences in reactivity between chains with end radicals of styrene and MAnh. MAnh-terminated growing chains do not react with MAnh monomers, but they almost exclusively react with styrene monomers (Alfrey and Lavin 1945; Hill et al. 1985). Therefore, the maximum content of MAnh units that can potentially be reached in SMAnh copolymers is 50 mol%. Only in this particular case a polymer with almost perfectly alternating building blocks can be obtained, by mixing styrene and MAnh monomers in a 1:1 molar ratio. This is not possible when styrene is present in excess, because styrene-terminated chains are capable of reacting with both styrene and MAnh monomers, the reaction with MAnh monomers being strongly favored (Alfrey and Lavin 1945; Deb and Meyerhoff 1984). If synthesis is performed in a batch-wise manner, this would result in polymers in which the overall ratio of styrene to maleic anhydride will be the same as the starting ratio, but in which the sequence distribution along the polymer chain may vary significantly: some segments will almost completely consist of alternating polystyrene and MAnh units, and others will have a high polystyrene content (Klumperman 2010; Montaudo 2001). In order to minimize this heterogeneity, the polymerization of SMAnh is typically performed in a continuous manner in which the monomer ratio is controlled by the continuous feed of monomers and the simultaneous collection of polymer product to create a steady-state condition during polymerization (Yao et al. 1999). In this way, the composition of the collected polymer reflects the monomer composition in the reactor and SMAnh polymerizes in a statistical manner, yielding a much more homogenous distribution of monomer units along the polymer chain (Fried 2003; Klumperman 2010). However, even under such steady-state conditions the synthesis of SMAnh leads to a rather inhomogeneous distribution of polymer chains differing in length and composition instead of well-defined molecules with a unique architecture and molecular weight. It is not clear yet whether or how this heterogeneity affects the membrane-solubilizing properties of SMA.

### Hydrolysis of styrene–maleic anhydride to form styrene–maleic acid

The use of SMA in the solubilization of lipid membranes and formation of nanodiscs is not based on the anhydride form SMAnh, but on the hydrolyzed acid form SMA (Fig. 2, Reaction 2). When SMAnh is mixed with water or alkaline solution, its anhydride units will be converted to the acid form with two carboxyl groups that become partly deprotonated, yielding water-soluble SMA. Hydrolysis of SMAnh is relatively slow due to the hydrophobic character of the polymer, but it may be accelerated by (1) using the anhydride as powder instead of granulate, (2) elevating the temperature, and (3) adding base (KOH or NaOH) during the reaction.

After hydrolysis, the SMA solution can be processed or purified in different ways. If a minimal amount of base has been used, the required pH can usually be obtained just by addition of extra base or acid. When an excess of base has been used, one can bring the SMA solution into a desired buffering environment either by using dialysis (Knowles et al. 2009) or by making use of their insolubility at low pH (Scheidelaar et al. 2015). In the latter case, SMA can be precipitated by addition of excess hydrochloric acid (HCl). After several washing steps with diluted HCl, the polymer can then be dried by lyophilization causing the residual HCl to evaporate, yielding fully protonated SMA. This can be dissolved in water and the pH can be readily adjusted by the addition of KOH or NaOH.

### pH-dependent properties of the styrene–maleic acid copolymer

SMA has amphipathic properties due to the hydrophobic styrene units and the hydrophilic carboxyl/carboxylate ([COOH]/[COO^−^]) groups. The degree of hydrophobicity depends not only on the ratio of styrene and maleic acid units in the polymer itself, but also strongly on pH. The two carboxyl groups in a maleic acid unit have different p*K*_a_ values: the first p*K*_a_ is close to 6, whereas the second one is close to 10 (Banerjee et al. 2012). This implies that at low pH, SMA essentially is non-charged, at neutral pH most of the maleic acid units will carry a single negative charge, and at high pH the maleic acid units will be charged at both carboxyl groups.

This pH dependence has major consequences for the conformation and solubility of SMA, as has been described for several other amphipathic polymers (Henry et al. 2006; Tonge and Tighe 2001). In the case of SMA, at neutral and high pH, electrostatic repulsions between the carboxylate groups dominate the hydrophobic effect and the polymer adopts a random coil conformation that dissolves relatively easily in aqueous solution. A decrease of the pH well below the lower p*K*_a_ of the maleic acid unit will lead to complete protonation of SMA. Charge repulsion is then lost and the hydrophobic effect causes SMA to adopt a globular conformation and eventually to precipitate as aggregates (Sugai and Ebert 1985; Sugai et al. 1982; Tonge and Tighe 2001). The exact pH range that mediates this structural transition will depend on the composition of the polymer and also on the ionic strength in the solution.

### SMA variants in membrane research

In all available studies on SMA and lipid membranes, the copolymers used had a ratio of styrene to maleic acid of 2:1 or 3:1, with an *M*_w_ in the range of 7.5–10 kDa. Systematic studies on the effects of SMA composition or molecular weight distribution have not been reported yet. For membrane solubilization, the polymers are commonly used at a pH between 7 and 8, at which values they will adopt a random coil conformation and the balance between the hydrophobic effect and electrostatic interactions will be optimal for interactions with lipid membranes.

For particular applications, SMAnh copolymers can also be covalently modified at the highly reactive anhydride moiety. In addition to hydrolysis to form SMA, this enables the introduction of different functional groups, via covalent binding in the form of esters, amides, or imides (see e.g., Henry et al. 2006). In this way, a large variety of SMAnh-based copolymers can be realized, each with its own properties and potential applications in membrane research, as will be discussed later.

## Interactions of SMA copolymers with lipid model membranes

In order to optimize the use of SMA copolymers in membrane research, different studies have focused on the physico-chemical characterization of SMALPs and on the solubilization of lipid vesicles by SMA. Model membranes are useful tools for such studies because they allow systematic variation of a wide range of lipid parameters, and they allow the formation of SMA–lipid particles with a well-defined lipid composition for detailed biophysical characterization. Here, we will give an overview of studies on the interaction of SMA with model membranes, starting with the characterization of SMALPs.

### Molecular structure and properties of styrene–maleic acid–lipid particles

The properties of SMALPs have been studied with a variety of biophysical techniques. The most common approaches to analyze the size of these nanodiscs include electron microscopy (Jamshad et al. 2015b; Knowles et al. 2009; Orwick et al. 2012; Scheidelaar et al. 2015), size exclusion chromatography (Jamshad et al. 2015b; Scheidelaar et al. 2015), and dynamic light scattering (DLS) (Knowles et al. 2009; Orwick et al. 2012; Scheidelaar et al. 2015; Zhang et al. 2015). Reported sizes are on the order of 10 nm, with minor variations, but it has not yet been investigated in detail what determines the size of SMALPs. Lipid composition does not appear to be a critical factor (Scheidelaar et al. 2015), but it is possible that different sizes result from variations in environmental conditions such as pH, salt concentration, or from the use of SMA polymers with differing composition or length. It has also been reported that using relatively low SMA-to-lipid ratios may result in an increase of the size of the nanodiscs (Vargas et al. 2015; Zhang et al. 2015). It should be noted, however, that virtually all solubilization experiments described in the literature have been performed with an excess of SMA, under which conditions the final size of nanodiscs is expected to be independent of SMA concentration. It is under these conditions that the physico-chemical properties of SMALPs have been characterized, as described below.

**Fig. 3:**
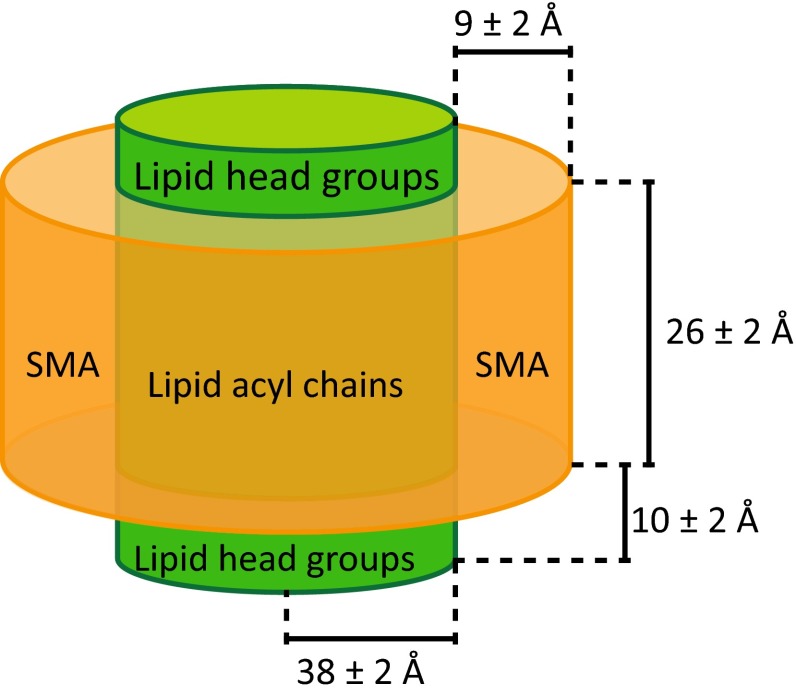
Dimensions of styrene–maleic acid/lipid particles (SMALPs) consisting of DMPC lipids and a SMA copolymer with a styrene–maleic acid ratio of 2, as determined from small-angle neutron scattering experiments (figure adapted from Jamshad et al. 2015b)

Small-angle neutron scattering experiments on SMALPs derived from DMPC vesicles revealed that the particles are discoidal with a diameter of about ~10 nm and a thickness of ~4.6 nm (Fig. 3, Jamshad et al. 2015b), which corresponds well to the thickness reported for pure DMPC bilayers in the fluid phase (Nagle and Tristram-Nagle 2000). The SMA copolymer belt surrounding the lipids was estimated to be ~0.9 nm thick, suggesting the presence of only one layer of SMA molecules. The number of polymer layers required to cover the thickness of the hydrophobic core of the bilayer is not known. Preliminary experiments in our lab (Koorengevel, Scheidelaar and Killian, unpublished results) indicate that vesicles of longer lipids require larger amounts of SMA to form nanodiscs. This is not unexpected because a thicker hydrophobic core needs to be shielded from the aqueous solution. The exact amount of SMA associated with one SMALP is, however, difficult to determine experimentally because of the heterogeneity of the polymers in size and composition. It is also not yet clear whether all of the associated polymer material is involved in the stabilization of the disc or whether some parts are forming “floppy ends” that stick out into the solvent or perhaps transiently associate with the head group area.

The interactions between lipids and SMA in SMALPs have also been investigated by Fourier transform infrared spectroscopy and NMR (Jamshad et al. 2015b; Orwick et al. 2012). It was found that the phenyl groups of SMA intercalate between the lipid acyl chains perpendicular to the plane of the lipids, and that the carboxyl groups interact electrostatically with the head groups of lipids that reside in the outer layer of the nanodisc. In addition, electron paramagnetic resonance (EPR) experiments (Orwick et al. 2012) revealed that carbons at certain positions in the acyl chains are restricted in their motion, consistent with insertion of the polymer phenyl groups. More information on the bilayer character of the nanodiscs was obtained by differential scanning calorimetry (DSC). These experiments showed that the lipids display a typical melting behavior of lipid bilayers. However, the phase transition is broadened and the transition temperature is somewhat shifted (Jamshad et al. 2015b; Orwick et al. 2012), presumably due to the relatively small number of lipids participating in the transition and their interaction with SMA. This shift in transition temperature seems to be dependent on the copolymer variant used as indicated by comparison of the effects of a copolymer with a ratio of styrene to maleic acid of 2:1 (Jamshad et al. 2015b) with a more hydrophobic 3:1 copolymer (Orwick et al. 2012). However, the difference between copolymer types has not been systematically studied yet. Notably, it was possible to record multiple DSC scans of SMALPS, but not for MSP nanodiscs, demonstrating the high temperature stability of SMALPs (Jamshad et al. 2015b).

The findings described above indicate that the SMA copolymer indeed stabilizes a small patch of lipid bilayer by associating with its hydrophobic core thereby justifying the term nanodisc as introduced previously for lipid bilayers bounded by MSP.

### Kinetics of membrane solubilization by SMA

**Fig. 4:**
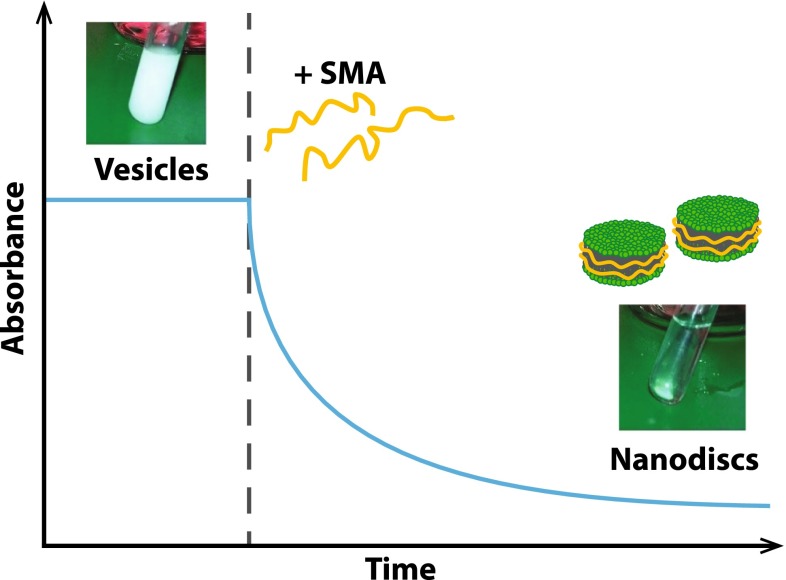
Schematic representation of a turbidimetry experiment. Suspensions of large lipid vesicles show a high degree of light scattering and appear milky. The addition of SMA leads to a clearing of the suspension due the formation of smaller nanodiscs. The concomitant rapid decrease in light scattering can be followed by measuring the apparent absorbance of the sample. For model membranes containing lipids in a fluid phase, this process typically occurs on time scales of minutes or tens of minutes (Scheidelaar et al. 2015)

The formation of SMA-bounded nanodiscs requires the solubilization of lipid membranes by the polymer. A simple and convenient way to monitor the kinetics of this process is turbidimetry (Scheidelaar et al. 2015). Lipid vesicles are large particles (hundreds of nanometers up to micrometers in size) and thus efficiently scatter UV light, whereas SMALPs are much smaller and scatter almost no light. Therefore, the solubilization process can generally be followed as a decrease in light scattering in time by using a spectrophotometer (see Fig. 4). This allows systematic studies on the effect of e.g., lipid composition, salt, or SMA concentration on the kinetics of solubilization.

**Fig. 5:**
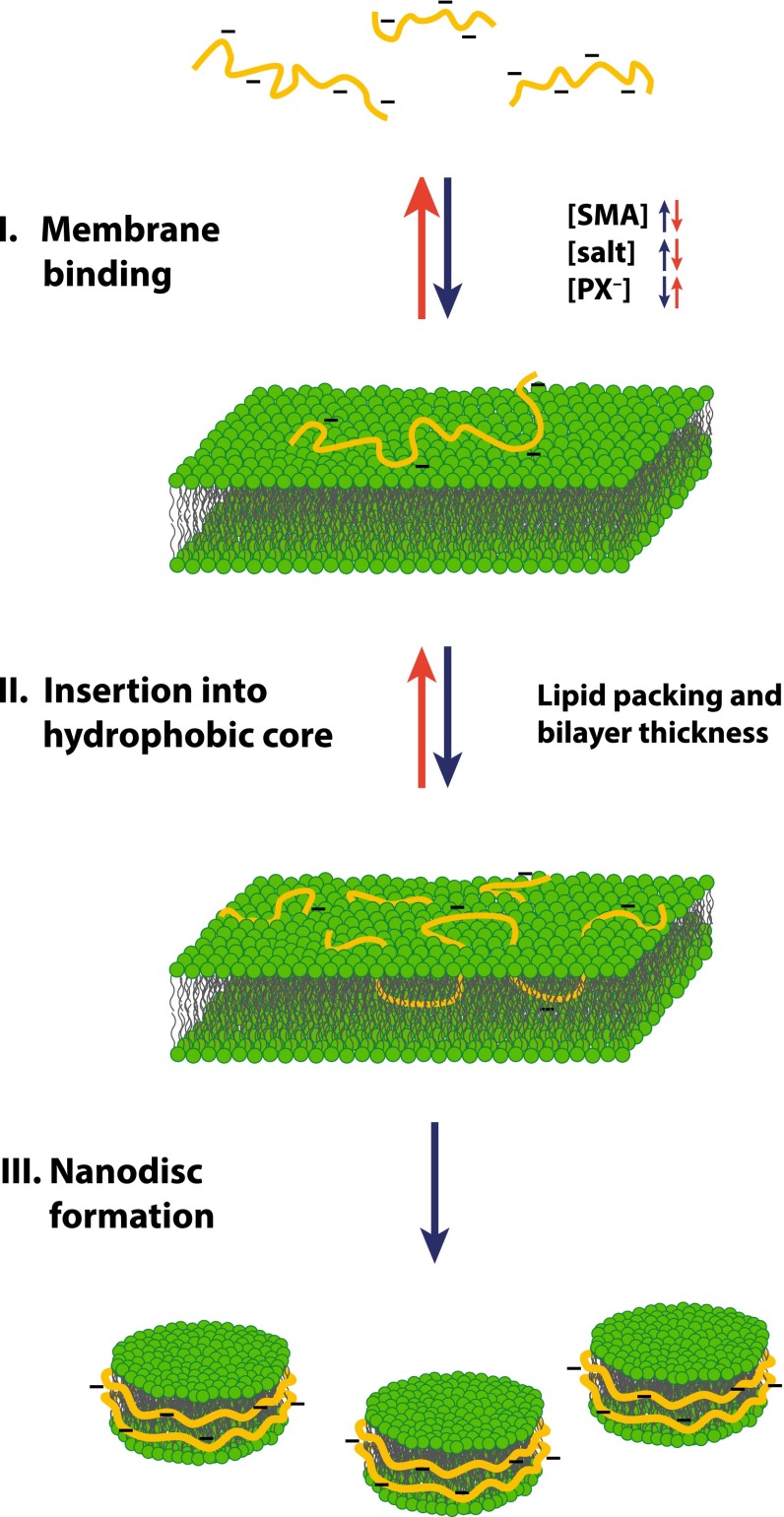
Three-step model for the solubilization of lipid membranes by SMA copolymers. Initially, SMA binds to the surface of the membrane (I), which is modulated by SMA and salt concentration and the presence of negatively charged lipids (PX^−^). The next step consists of the insertion of the polymer molecules into the hydrophobic core of the membrane (II), driven by the hydrophobic effect. This process is modulated by the lipid packing and bilayer thickness. Finally, the membrane is solubilized and nanodiscs are formed (III). The kinetics of the solubilization are determined mainly by the second step and after SMA has penetrated into the hydrophobic core of the bilayer, the formation of nanodiscs is a downhill process (see text for details, figure adapted from Scheidelaar et al. 2015)

Using this approach, it was found that many different physical parameters affect membrane solubilization by SMA and a three-step model was developed to describe its mode of action (Scheidelaar et al. 2015) (Fig. 5). The first step consists of the binding of SMA to the surface of the lipid bilayer. This process can be promoted by increasing the amount of SMA and can be further modulated by electrostatic interactions: the presence of anionic lipids causes repulsion and thus impairs binding of the negatively charged polymer, while increasing the ionic strength promotes binding. In the second step, SMA inserts into the hydrophobic core of the membrane. This is strongly affected by lipid packing (e.g., membrane fluidity and lateral pressure) and bilayer thickness, with both tight packing and thick membranes impairing penetration of SMA into the hydrophobic core of the lipid bilayer. The final step is the actual solubilization of the bilayer and the simultaneous formation of nanodiscs. The second and third steps are closely connected since nanodisc formation also is influenced by lipid packing and bilayer thickness, although in a different way. For example, for thicker membranes the free energy cost of breaking up the bilayer is larger, and lipid packing plays a role because in the nanodiscs the hydrophobic groups of the polymer will have to insert in between the hydrophobic chains of the lipids, which will be more difficult the more tightly the lipids are packed.

When sufficient polymer is bound to the membrane surface, the kinetics of the solubilization process are mainly determined by the second step. A subsequent insertion into the hydrophobic core of the bilayer then bears a large enthalpic penalty for the polar/charged carboxyl groups. Therefore, as soon as anionic SMA reaches the hydrophobic core of the bilayer, the thermodynamically highly favorable formation of nanodiscs will occur as a downhill process. More detailed thermodynamic considerations can, however, not be derived based on turbidimetry because it relies on size as the sole observable feature. Nevertheless, this assay led to the identification of major parameters that influence membrane solubilization by SMA.

Consistent with this model, it was found that the phase state of the lipids is an important factor for the efficiency of solubilization (Scheidelaar et al. 2015). In general, lipids in the gel phase are much more difficult to solubilize than the more loosely packed lipids in the liquid-crystalline phase. Solubilization of phosphatidylcholine bilayers with saturated acyl chains of a certain length thus shows a distinct temperature dependence that is strongly linked to the gel-to-liquid crystalline phase transition temperature of the lipid. The most rapid and efficient solubilization is generally obtained at this phase transition temperature, where large packing defects help the polymer to enter the hydrophobic core of the membrane. For lipids in the fluid phase, it has been shown that the efficiency of solubilization can be further modulated by changes in lipid packing. For example, it was found that introduction of unsaturated bonds leads to a decrease in the solubilization efficiency. This somewhat counterintuitive and unexpected observation was attributed mainly to an increase in lateral pressure in the hydrophobic core of the bilayer that hinders SMA insertion and thus membrane solubilization, as supported by experiments involving non-bilayer-forming lipids (Scheidelaar et al. 2015).

Despite the individual contributions of different physical properties of the lipids to the kinetics of solubilization, SMA does not appear to preferentially solubilize certain lipid species, i.e., nanodiscs bounded by SMA maintain the overall lipid composition of the vesicles as exemplified for a homogeneous lipid mixture that reflects the composition of *E. coli* inner membranes (Scheidelaar et al. 2015). This result implies that solubilization is mainly determined by the physical properties of the lipid membranes rather than by the properties of individual lipid species.

### Comparison of the mode of action of different solubilizing agents

The driving force for membrane solubilization by SMA and the formation of nanodiscs lies in the amphipathic properties of the polymer. The hydrophobic effect promotes the insertion of its apolar parts into membranes while the polar/charged carboxyl groups render the nanodisc soluble in an aqueous environment. These amphipathic properties are not unique for SMA copolymers however. Detergents, MSPs, and amphipols all exhibit a similar amphiphilicity. Yet, these molecules act very differently when mixed with lipid membranes. Detergents, for instance, dissolve bilayers completely, and generally form micelles instead of nanodiscs. MSPs on the other hand can form nanodiscs together with lipids, but they generally need to be reconstituted from mixtures with detergent (Bayburt et al. 2002). This is because MSPs are α-helical proteins and therefore relatively bulky. As a result, they only can insert into membranes and form nanodiscs when the lipids are at the gel-to-liquid crystalline phase transition, where lipid packing defects exist (Scheidelaar et al. 2015; Wan et al. 2011). Finally, amphipols generally have large hydrophobic groups that help to stabilize membrane proteins, but that make nanodisc formation unfavorable due to steric hindrance and loss of conformational entropy. In the case of SMA, very efficient formation of nanodiscs occurs, which has been attributed mainly to the small size and rigidity of its phenyl groups (Scheidelaar et al. 2015). These allow the polymer to efficiently insert into lipid bilayers with a minimal loss in conformational entropy and minimal intrinsic steric hindrance when wrapped around a nanodisc.

### Incorporation of membrane proteins in SMALPs

The structure of SMALPs and vesicle solubilization by SMA as described above have been studied in detail for SMALPs that consist of lipid material only. However, it is also possible to insert membrane proteins into SMALPs, as was initially shown for PagP and bacteriorhodopsin (Knowles et al. 2009; Orwick-Rydmark et al. 2012) and later for the potassium channel modulator protein KCNE1 (Sahu et al. 2013). To achieve this, proteins can either first be solubilized with detergent and then reconstituted into liposomes by conventional techniques or native membranes containing the protein can be supplemented with synthetic lipid after which SMA is added to obtain SMALPs (see e.g., Goddard et al. 2015). Importantly, it was found that the structure and activity of the proteins in SMALPs remain intact during the solubilization process and that, once incorporated into SMALPs, the proteins can be studied by a variety of methods, as will be discussed later.

## Solubilization of membrane proteins from cellular membranes

### Solubilization

**Fig. 6:**
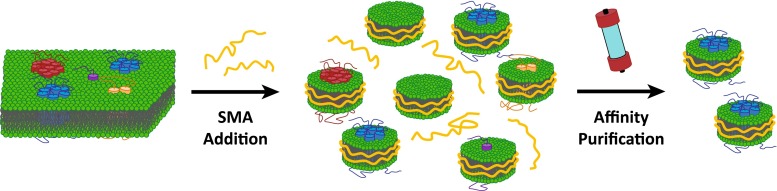
Extraction of membrane proteins with native lipid environment by SMA. SMA additions leads to the formation of native nanodiscs containing different MPs or only lipid material. Subsequent affinity purification allows for the isolation of native nanodiscs with the protein of interest

In a number of recent studies, it has been shown that SMA polymers can extract membrane proteins directly from intact membranes of cells and organelles without addition of conventional detergent (see Fig. 6). The biological sources include bacteria (Dörr et al. 2014; Paulin et al. 2014; Postis et al. 2015; Prabudiansyah et al. 2015; Swainsbury et al. 2014) and yeast (Gulati et al. 2014; Jamshad et al. 2015a; Long et al. 2013; Skaar et al. 2015) as well as cultures of insect (Gulati et al. 2014) and human cells (Gulati et al. 2014; Jamshad et al. 2015a), which together account for all major biosystems that are used for recombinant MP production. The proteins that have thus been incorporated into native nanodiscs span a wide variety of MPs of different sizes (see e.g., forthcoming Table 1), ranging from those with a single membrane spanning α-helix (Paulin et al. 2014) to oligomeric complexes comprising up to 36 transmembrane helices (Postis et al. 2015). The extraction of MPs in native nanodiscs hence seems to be independent from the host membrane and to be generically applicable to all members of this class of proteins. Furthermore, MPs can be directly extracted in these stable particles from purified membrane fractions (Gulati et al. 2014) or whole cells (Dörr et al. 2014) and the reported solubilization yields using SMA are generally comparable with those of established detergent-based protocols with deviations in both directions.

Some MPs are relatively difficult to solubilize from native membranes, which can be due to e.g., a low lipid/protein ratio or tight lipid packing. For such proteins, a potential strategy to improve incorporation into nanodiscs might be the addition of synthetic DMPC lipid. This lipid is very efficiently solubilized by SMA (Scheidelaar et al. 2015) and it has been used in early studies for assisted solubilization of proteins from native membranes (Knowles et al. 2009; Orwick-Rydmark et al. 2012). Reconstitution into DMPC liposomes also allowed solubilization of a protein of the β-barrel type (PagP) by SMA (Knowles et al. 2009). However, the solubilization of β-barrel proteins directly from the outer membranes of bacteria, chloroplasts, or mitochondria has not yet been reported. Even so, the variety of successfully incorporated proteins into nanodiscs bounded by SMA indicates a general applicability of SMA isolation for MPs of any class.

### Purification

Once solubilized in the form of native nanodiscs, the protein of interest can be purified using standard methods. For instance, size exclusion chromatography can be used to isolate native nanodiscs with functional MP complexes of high natural abundance (Long et al. 2013). Furthermore, purifications to homogeneity can be achieved by exploiting the Ni^2+^ affinity of His-tagged proteins (Dörr et al. 2014; Gulati et al. 2014; Jamshad et al. 2015a; Swainsbury et al. 2014) or binding of antibodies (Gulati et al. 2014). Both of these approaches render protein yields and purities that are similar to those obtained by detergent-based protocols. However, there is also a potential problem with Ni^2+^-affinity purifications. Based on the physico-chemical properties of SMA, several interactions are possible that may impair His-tag binding in the presence of SMA in solution and/or associated to nanodiscs. These include electrostatic interactions between His tags and SMA due to the anionic character of the polymer, binding of the phenyl groups of SMA to His tags, and occupation of the free coordination sites of immobilized Ni^2+^ by the carboxylates in SMA. Possibilities to improve purification yields in this case include removal of excess SMA by prior filtration, decreasing the amount of SMA used for solubilization, increasing the amount of available Ni^2+^, increasing the number of histidines in the tag sequence, or simply diluting the solution before addition of Ni^2+^-containing material to decrease the effective concentration of excess SMA. Nothing has been reported so far on the compatibility with other purification systems (Strep tag, FLAG tag, etc.), but also in those systems inhibitory effects on binding might occur, with similar possibilities to improve the yields. Thus, it is likely that native nanodiscs with any MP can be purified without major difficulties by using the established protocols for these particles.

### Stability

Low protein stability is a common problem in detergent-based methods for membrane protein isolation and purification. Especially for proteins that are challenging in this respect, the incorporation into native nanodiscs can be an important advantage. As a prominent example, proteins of the family of G-protein coupled receptors are notoriously difficult to study since they have a high intrinsic flexibility and are thus unstable in detergent micelles (Rasmussen et al. 2007). Yet, also a member of this family (human A_2A_R) has been successfully isolated using SMA polymers (Jamshad et al. 2015a). Many other reports also suggest a superior stability of proteins in native nanodiscs as compared to those solubilized in detergent (Dörr et al. 2014; Gulati et al. 2014; Knowles et al. 2009; Swainsbury et al. 2014). Other advantages of nanodiscs over detergent micelles are an improved storage potential (Dörr et al. 2014; Jamshad et al. 2015a) and a less dynamic environment that does not require a pool of soluble polymer molecules to maintain particle stability. Proteins in native nanodiscs thus essentially behave like soluble proteins and are amenable to standard biochemical and biophysical analysis.

### Characterization of native nanodiscs

The size of native nanodiscs incorporating MPs is fairly uniform for each individually studied protein, but diameters vary between 10 and 24 nm for different proteins. Although many reports suggest that the presence of incorporated proteins increases the particle size (Knowles et al. 2009; Long et al. 2013; Orwick-Rydmark et al. 2012), a comparison of different studies does not show a clear systematic correlation of the disc size and the dimensions of the membrane-spanning domain of the protein (see Table 1). For instance, nanodiscs containing a KcsA tetramer with eight transmembrane (TM) helices have an average size of 10 nm, whereas the smaller bacteriorhodopsin (7 TM helices) yields slightly bigger particles with diameters of 12 nm.

**Table 1:** Particle size of native nanodiscs and SMALPs with different incorporated proteins

Protein and native organism	No. of membrane-spanning segments	Estimated particle diameter	Methods
AcrB (trimer) (Postis et al. 2015) *Escherichia coli*	36 (3 × 12) helices	14 nm^a^	TEM
Respiratory complex IV (Long et al. 2013) *Saccharomyces cerevisiae*	26 helices^b^	12 nm	TEM
P-glycoprotein 1 (Pgp) (Gulati et al. 2014) *Homo sapiens* (expressed in *Trichoplusia ni* cells)	12 helices	10–15 nm^c^	TEM
Photosynthetic reaction center (Swainsbury et al. 2014) *Rhodobacter sphaeroides*	11 helices plus hydrophobic chromophores	13–14 nm	TEM, DLS
KcsA (Dörr et al. 2014) *Streptomyces lividans* (expressed in *E. coli*)	8 helices	10 nm	TEM
Bacteriorhodopsin (Knowles et al. 2009; Orwick-Rydmark et al. 2012) *Halobacterium salinarum* (solubilized after DMPC addition)	7 helices	12 nm	DLS
PagP (Knowles et al. 2009) *E. coli* (solubilized from DMPC liposomes)	8 strands	11 nm	TEM, DLS
PBP2 and PBP2a (complex) (Paulin et al. 2014) *Staphylococcus aureus*	Undefined^d^	18–24 nm^d,e^	TEM

^a^Not explicitly stated in reference

^b^Possible variations depending on complex stoichiometry: the core subunits of yeast Complex IV (Cox I, II and III) contain 21 TM helices, supernumerary subunits contribute an additional 5; 14 TM helices (Cox I and II) are essential components for activity

^c^Deviations due to large flexible soluble domains; diameter of nanodisc probably corresponds to lower value of 10 nm

^d^Unpurified soluble fraction containing MPs of different size

^e^Size varies with cell line

In some of the studies referred to in Table 1, the ratio of lipid to protein in native nanodiscs was determined and also here a comparison of different proteins reveals considerable variations. A photosynthetic reaction center was isolated and purified together with 150 lipid molecules from bacterial membranes (Swainsbury et al. 2014) whereas only 11 lipid molecules were coextracted with PagP (Knowles et al. 2009) and 40 with AcrB (Postis et al. 2015), respectively. Note that neither of the latter low values would be in accordance with a full annular ring of lipids around the incorporated protein and thus some of the hydrophobic surface of the protein would be in direct contact with the polymer. The low-resolution EM structure of Pgp also lacks a large lipid belt, which supports the view of a limited amount of bound lipid (Gulati et al. 2014). In other cases, relatively large lipid/protein ratios were estimated based on the ratio in reconstituted proteoliposomes (Orwick-Rydmark et al. 2012) or on theoretical considerations assuming 180–200 lipid molecules in protein-free SMALPs some of which would be replaced by the protein (Long et al. 2013). However, given the huge variations in experimentally determined lipid/protein ratios and in view of the considerable differences in nanodisc size such estimations may perhaps not be very reliable.

There are several other potential explanations for the large variation in diameter and protein/lipid ratios in native nanodiscs. For example, low amounts of lipids could originate from the formation of oligomeric complexes of the incorporated proteins. Furthermore, variations in experimental conditions, such as the SMA/lipid ratio used for initial solubilization could affect the composition and size of native nanodiscs. Systematic studies on this with biological membranes have not been reported yet, but the heterogeneity of SMA, the presence of structurally different MPs and differences in membrane lipid composition will result in further deviations between samples. Thus, many different parameters will probably influence whether or not a full annular ring of lipids is coextracted with the protein or whether there are alternating polymer and lipid contacts with the hydrophobic surface of the protein.

For all systems in which the lipid content of native nanodiscs has been investigated, it was demonstrated that at least several native lipid molecules are coisolated. Similar to the situation in protein-free SMALPs, these lipid molecules are likely to be organized in a limited version of a bilayer (Jamshad et al. 2015b; Orwick et al. 2012), which makes native nanodiscs the only system that is capable of extracting MPs out of the membrane while conserving their native environment. This is supported by several reports showing that the lipid composition in solubilized nanodiscs is very similar to the composition of the membrane they were extracted from (Dörr et al. 2014; Long et al. 2013; Prabudiansyah et al. 2015; Swainsbury et al. 2014). This (near) native environment—whether organized in a genuine full bilayer or not—is likely the major reason for the higher stability of MPs in native nanodiscs.

### Native interactions of membrane proteins with lipids

An important implication of the conservation of a native environment around an MP in native nanodiscs is the possibility of direct biochemical analysis of native interactions of the protein with surrounding lipids or with other membrane components. For example, for the potassium channel KcsA (Dörr et al. 2014) and the bacterial translocon SecYEG (Prabudiansyah et al. 2015), it was found that the composition of the lipids in the purified native nanodiscs was significantly different from that of the bulk membrane or the total solubilized fraction. In both cases, anionic lipids were enriched in the native nanodiscs containing the respective proteins, consistent with functional relevance of anionic lipids for these proteins as deduced by model membrane studies.

It should be noted here that the copurification of a larger number of (annular) lipids in a stable complex in native nanodiscs is distinctly different from coextraction of lipids with MPs in detergent-based methods. In nanodiscs, part of the native lipid environment is retained, while detergent extraction generally results in a selective copurification only of those lipids that are tightly bound to the target protein (Shinzawa-Itoh et al. 2007; Valiyaveetil et al. 2002). Examples of the copurification of more extensive lipid material also exist, but this process is strongly dependent on the detergent used (Ilgü et al. 2014). Moreover, due to the dynamics in the micellar organization, such complexes of lipids with the protein in detergent will likely not be stable in time, thus leading to a biased picture of the situation in biomembranes.

### Native interactions of membrane proteins with other proteins

A further application of the extraction of MPs in native nanodiscs is the study of interactions between different proteins, since MPs whose membrane-embedded parts are closely interacting can be captured together in the same nanodisc. This readily allows the isolation of stable homooligomers of MPs (Dörr et al. 2014; Gulati et al. 2014; Postis et al. 2015) and large functional MP complexes (Long et al. 2013; Swainsbury et al. 2014), but can also be exploited for investigations of more dynamic interactions. For example, the detergent-labile binding of two PBP proteins could be confirmed by an approach using coimmunoprecipitation after solubilizing bacterial cells with SMA (Paulin et al. 2014). More recently, the purification of the SecYEG channel as functional complex with an interacting peripheral membrane protein in native nanodiscs was reported. This provided information on the interactome of the channel, which could not be obtained in detergent-based approaches due to the detergent sensitivity of the complexes (Prabudiansyah et al. 2015). Native nanodiscs thus serve as a tool to study preferential interactions of the MP of interest with both lipids and other proteins.

## Structural and functional investigations of proteins in native nanodiscs or SMALPs

### Membrane protein structure

Native nanodiscs are small soluble particles that readily allow structural characterization of the incorporated proteins by solution-based techniques. The small size of the nanodiscs is highly advantageous because of the low degree of light scattering, facilitating the use of optical techniques like circular dichroism (Dörr et al. 2014; Gulati et al. 2014; Jamshad et al. 2015a; Knowles et al. 2009) and fluorescence spectroscopy (Dörr et al. 2014; Gulati et al. 2014; Prabudiansyah et al. 2015) as well as absorption measurements in the UV and visible light range (Long et al. 2013; Swainsbury et al. 2014). The strong structural resemblance of nanodiscs bounded by SMA and MSP should, in principle, make native nanodiscs (and also MPs reconstituted into synthetic SMALPs) suitable for the complete range of methods that has been established for MSP nanodiscs (Bayburt and Sligar 2010; Schuler et al. 2012). One notable advantage of the use of SMA is that it exhibits optical properties that differ from those of proteins. This is of particular relevance for far-UV circular dichroism spectroscopy where the MSP exhibits a strong α-helical signal that will be superimposed on that of the protein of interest. Although SMA does absorb in the UV range the amount of polymer material associated with nanodiscs is low enough to allow the acquisition of high-quality spectra (Dörr et al. 2014). Similarly, intrinsic tryptophan fluorescence of proteins in native nanodiscs is not affected by the presence of SMA.

Another technique that has been successfully used to characterize membrane proteins in SMALPs is EPR spectroscopy (Orwick-Rydmark et al. 2012; Sahu et al. 2013, 2014). This allowed for accurate distance measurements that revealed similar dynamics of bacteriorhodopsin as compared to its state in the plasma membrane (Orwick-Rydmark et al. 2012). It was further shown that proteins in SMALPs render data of better quality than those obtained in liposomes (Sahu et al. 2013). Studies on protein structure involving the methodologically related NMR spectroscopy are, however, still lacking. This could be due to the particle size of nanodiscs, which is rather close to the limitations of solution-state NMR. However, both solution- and solid-state NMR approaches have been established for MSP nanodiscs (Ding et al. 2015) and therefore, in principle, should also be possible for proteins in native nanodiscs. An alternative NMR approach using aligned systems, as reported for relatively large bicelles (Howard and Opella 1996), unfortunately does not appear to be feasible, since neither standard MSP-nanodiscs nor SMALPs tend to align in magnetic fields: in both cases isotropic peaks in ^31^P NMR are observed that suggest fast reorientation of the particles in all directions (Park et al. 2011; Vargas et al. 2015; Zhang et al. 2015).

The single-particle character of nanodiscs bounded by either SMA or MSP also makes them promising targets for structural investigation by electron microscopy (EM), as demonstrated for example for MSP nanodiscs with the anthrax pore toxin (Katayama et al. 2010) and the ribosome-SecYE complex (Frauenfeld et al. 2011). The EM field has recently undergone a drastic transition due to substantial improvements in both experimental equipment and data analysis software (Bai et al. 2015a). This now results in electron density maps of a quality that allows the de novo-determination of structures even of relatively small proteins (MW < 200 kDa) at (near) atomic resolution (Bai et al. 2015b). The fact that neither crystals nor large amounts of purified proteins are required in single-particle EM methods make this approach particularly interesting for MPs and their complexes. Up till now, negative-stain transmission EM has been widely used as a general tool to determine the size of nanodisc particles (see Table 1). In some cases, images from both negative stain and cryo-EM have been further processed and aligned to yield low-resolution 3D structures of the proteins in the nanodiscs, as was reported for AcrB (Postis et al. 2015) and the ABC transporter Pgp (Gulati et al. 2014). The structures were in good agreement with available X-ray structures of these proteins (or their homologues) and thus serve as a good first approximation for the structure of MPs in their native state. High-resolution structures for proteins in native nanodiscs have not been reported to date, but cryo-EM might be a suitable technique to achieve this. This approach is particularly promising for large MPs or for MP complexes that are relatively unstable in detergent. In combination with complementary techniques like solid-state NMR spectroscopy that allow the study of protein dynamics, native nanodiscs could thus become a powerful platform for ex vivo structural biology of MPs.

### Membrane protein function

Biophysical characterization of MP function in nanodiscs largely benefits from the fact that the soluble domains of the protein on either side of the membrane are accessible to the solvent. Thus, addition of solutes can be studied while a native-like environment is conserved. Indeed, binding studies with small molecules have been effortlessly performed with proteins in native nanodiscs using radioactive ligands (Gulati et al. 2014; Jamshad et al. 2015a) or detection of binding by fluorescence quenching (Gulati et al. 2014). Furthermore, native nanodiscs have been shown to be suitable for other assays where protein activity was tested using NMR (Knowles et al. 2009) and fluorescence spectroscopy (Prabudiansyah et al. 2015) as well as absorbance measurements (Long et al. 2013; Swainsbury et al. 2014). In general, the activity of the proteins investigated in native nanodiscs is either similar to that in detergent micelles or even improved, again indicating the superiority of the conserved (native) lipid environment over detergent. Together with the generally unperturbed protein structure, it can thus be assumed that both the native structure and function of MPs are conserved in native nanodiscs.

One should note, however, that the use of SMA also has potential limitations. For instance, the higher order of lipids in SMALPs as determined for particles derived from model membranes (Orwick et al. 2012) could cause a higher rigidity of the lipid environment. Apart from a favorable increase in protein stability this could also hinder conformational transitions or helical movement and thus interfere with protein function in native nanodiscs. Furthermore, the presence of SMA could impair binding studies involving the use of (multiple) positively charged molecules, because the high negative charge density of SMA can interfere with binding of these molecules to their target proteins. Also, the fact that proteins in nanodiscs are accessible to the same solvent on both sides of the membrane is not necessarily always an advantage. It is for example incompatible with functional studies on vectorial transport of small molecules by ion channels or transport proteins, since such assays usually require compartment-forming systems. One way to resolve this is the reconstitution of the protein directly from native nanodiscs into such a system. Promising results indicating that this is feasible were recently reported for the channel-forming protein KcsA (Dörr et al. 2014). Native nanodiscs containing this protein were added to a planar lipid bilayer system, resulting in spontaneous insertion of the protein into the bilayer, which allowed electrophysiological characterization of the channel and validation of its functionality. This proof-of-principle example of a direct reconstitution of MPs from native nanodiscs into compartment-forming bilayers opens the door to a wide range of potential further applications.

## Summary and outlook

Recent reports on the use of SMA polymers have highlighted various applications of these versatile molecules to study membranes and membrane proteins. This shows that the system is rapidly gaining acceptance in the field. However, current use of the technique is still far from exploiting its full potential. Here, we will review some possibilities offered by current methods of SMA extraction and we will discuss some new applications of SMA that may become available in future membrane research.

### Applications of native nanodiscs and new possibilities offered by transfer to other environments

**Fig. 7:**
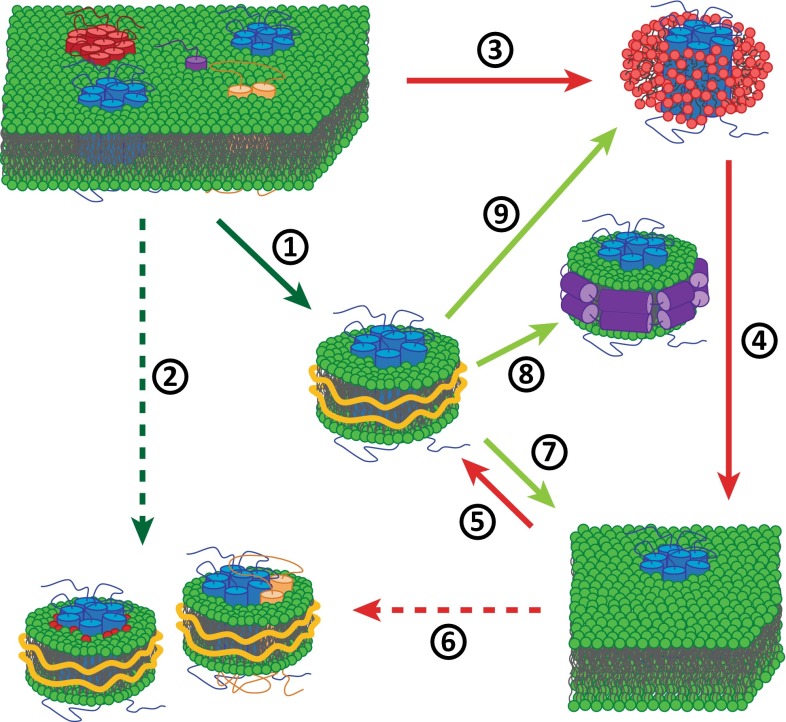
Different applications of native nanodiscs. *Dark green arrows* indicate conservation of the native environment, *light green arrows* display possibilities for the transfer to controlled environments. *Red arrows* display approaches that generally use synthetic environments. The *dashed arrows* represent approaches in which complexes of MPs with specific lipids (*red*) or other proteins (*yellow*) can be isolated from native (2) or synthetic (6) environments

Figure 7 illustrates established and potential future applications of membrane solubilization by SMA. The various applications are depicted as numbered arrows in the figure, which will be referred to in the text. In the following paragraphs, we will zoom in on the possibilities associated with each of the different arrows.

*Arrow 1* As discussed extensively already, the isolation of membrane proteins in native nanodiscs offers exciting possibilities for their structural and functional characterization in an ex vivo approach, i.e., MPs are extracted directly from intact cells while conserving a native environment. Native nanodiscs are readily amenable to single particle techniques right after purification and can thus be studied by many biophysical techniques. EM in particular is a promising technique for structure determination of MPs in native nanodiscs. Given a sufficient sensitivity of any technique employed, it would even be possible to study isolated MPs and their complexes at endogenous levels of expression. These properties make native nanodiscs a promising tool that will likely develop into a powerful platform for structural and functional characterization of MPs.

*Arrow 2* involves the copurification of lipids and other proteins with MPs in the same nanodisc. The results reported to date support the assumption that SMA-based extraction renders a snapshot view of an MP in its natural context, allowing determination of preferential lipid–protein and protein–protein interactions (Dörr et al. 2014; Paulin et al. 2014; Prabudiansyah et al. 2015). Combined approaches involving different pull-down assays and separation techniques on native nanodiscs may thus provide a convenient tool to obtain detailed information on the interaction profile of MPs. The solubilization of several proteins in one native nanodisc could then be considered a form of noncovalent “mild crosslinking”.

*Arrows 3–5* SMA can also be applied to model membranes (arrow 5) in which proteins are reconstituted in the conventional way by using detergent (arrows 3 and 4). This allows the preparation of SMALPs containing an MP embedded in a defined lipid environment. The big advantage would be that the lipid composition can be systematically varied in a similar way as has been established for MSP-bounded nanodiscs (Schuler et al. 2012).

*Arrow 6* SMALPs derived from synthetic bilayers of specific lipid and/or protein composition can also be used as a tool to analyze preferential interactions of MPs with lipids or other proteins. The underlying principle is similar to that already presented for native nanodiscs, but the advantage is that the interactions can be studied in a more controlled way. For example, hydrophobic matching preferences could be assayed by reconstituting proteins into bilayers composed of two lipids with acyl chains of different lengths and by subsequently monitoring whether one of the lipids is enriched in isolated and purified protein-containing SMALPs. By contrast, traditional approaches to investigating such interactions are cumbersome and usually require labeled molecules that are detected indirectly by biophysical techniques (Lee 2011). It is also possible to reconstitute the proteins into bilayers at different lipid/protein ratios and then analyze the protein content of the purified nanodiscs. This would allow straightforward studies on protein–protein interactions and oligomerization processes and enable investigations on how they depend on, for instance, the lipid environment.

*Arrow 7* A limitation of native nanodiscs is that they may be incompatible with intended downstream applications. Examples are techniques that require the presence of separate compartments such as transport assays and electrophysiology. In those cases, it would be convenient to reconstitute the protein from the native nanodisc environment directly into synthetic bilayers, as was recently reported for the channel protein KcsA (Dörr et al. 2014). MPs treated this way may thus be studied in a controlled and compartmentalized environment without ever being destabilized in detergent. It should be noted however that in this example of KcsA the successful reconstitution of a single channel would be sufficient for functional measurements. A big challenge still remains in the quantitative reconstitution of the entire native protein material including surrounding lipids. This could have important implications because it might enable structure determination by X-ray crystallography of membrane proteins in a near-native lipidic environment. Such reconstitution is not straightforward however, especially because of the high affinity of SMA for lipids, which complicates their removal and may impair the formation of extended bilayers. It has been suggested that lowering the pH would solve this problem (Jamshad et al. 2011). However, in our hands this approach was not successful (Koorengevel, Scheidelaar and Dörr, unpublished observations). Reconstitution via a decrease in pH is likely problematic because the increased hydrophobicity of a protonated polymer causes precipitation of the nanodiscs together with enclosed lipids and proteins. Nevertheless, it is an intriguing idea to exploit the pH dependence of SMA to destabilize nanodiscs. For instance, addition of excess lipid may be sufficient to facilitate reconstitution in bilayers using this approach.

Reconstitution from native nanodisc into bilayers or other membrane mimicking environments may also be important for studies in which specific properties of SMA are interfering with the analysis. For instance, measurements at low pH or assays that require high amounts of Mg^2+^-stabilized nucleotides may be a problem due to destabilization of the nanodiscs by protonation or chelated divalent cations, respectively. Furthermore, it is possible that insertion of the phenyl groups between the lipid chains induces changes in lipid packing in nanodiscs that may be unfavorable, or that the presence of phenyl groups is a disadvantage in processes involving cation–π or π–stacking interactions.

*Arrows 8 and 9* As discussed above, there are several reasons why native nanodiscs may be incompatible with the assays or methods to be used. In such cases purification and stabilization of an MP in native nanodiscs and further transfer into other membrane mimics may be a convenient approach. Depending on the applications, this environment may be compartment-forming systems, as discussed above, or it could be MSP nanodiscs or detergent micelles. In particular, transfer to MSP nanodiscs could be convenient because many applications already have been developed and tested for these particles. However, reconstitution directly from native nanodiscs into MSP nanodiscs has not been reported yet. Similarly, it could be advantageous to purify proteins in the form of native nanodiscs and replace the nanodiscs for detergent prior to functional or structural studies. This is the case for example when a protein is not stable enough in detergent for purification, but the analysis method is incompatible with nanodiscs bounded by SMA.

### Preferential solubilization of membrane domains by SMA

Preferential solubilization of membrane domains by SMA may be another useful application for future membrane research. Although the polymer is promiscuous with respect to solubilization of phospholipid species (Dörr et al. 2014; Long et al. 2013; Prabudiansyah et al. 2015; Scheidelaar et al. 2015; Swainsbury et al. 2014), it does solubilize certain types of membranes more easily than others. For example, membranes with very low lipid/protein ratios will be relatively difficult to solubilize. This was exploited in a recent study on plant thylakoid membranes to prepare a non-solubilized fraction that was enriched in certain MP complexes (Bell et al. 2015). As another example, membranes with lipids in a fluid phase are more efficiently solubilized than membranes with gel-phase lipids (Scheidelaar et al. 2015). This may be exploited to selectively solubilize the fluid-phase lipids in membranes that exhibit phase separation. The same could hold for membranes containing liquid-ordered domains enriched in sphingolipids and cholesterol (often termed “lipid rafts”). In view of their tight packing, these domains are likely to be more resistant against solubilization by SMA than the more fluid phosphatidylcholine-rich domains, leading to a selective solubilization of the liquid-disordered domain (Dominguez and Killian, manuscript in preparation). Thus, SMA could provide an alternative means of purifying liquid-ordered domains without the need for conventional detergent. This would be a potential advantage over established detergent-based methods that can affect the phase behavior of the system (Heerklotz 2002).

### SMALPs as membrane mimics to study membrane interactions with water-soluble proteins and peptides

An alternative application of nanodiscs bounded by SMA or MSP is their use as small soluble membrane mimics for any kind of study on membrane interaction of water-soluble peptides or proteins. Due to their limited size, nanodiscs may be particularly useful for studying initial stages in processes that involve membrane-mediated peptide oligomerization. No such studies have been reported yet, but it is possible that nanodiscs are suitable to study early stages of pore formation in membranes by oligomeric species of amyloid-forming proteins or by pore-forming antibiotic peptides. The same holds for amyloid aggregation at a membrane surface, where the small available surface area on nanodiscs may be exploited to trap smaller oligomeric intermediates.

### Reduction of damage from autocatalytic processes

Nanodiscs bounded by SMA or MSP exhibit still another advantage: because of their size limitations these particles are less prone to damage by autocatalytic processes that have the characteristics of chain reactions than continuous bilayer systems. For instance, light-induced oxidation processes may corrupt all molecules in a damaged liposome, whereas in nanodiscs the same process will affect much fewer lipids and generally only one protein (Swainsbury et al. 2014). This principle may be important for fundamental studies on effects of ionizing radiation on lipids and membrane proteins, but it may also be important for potential future applications, for example the construction of solar cells using photosynthetic membrane proteins and lipids.

### SMA as acceptor system for cell-free protein production

SMALPs may possibly also find a use as acceptor bilayer system for cell-free MP production, as was shown for nanodiscs bounded by MSP (Roos et al. 2012). So far, this has not been reported but advantages over the MSP-bounded discs would be that the preparation of SMALPs is cheaper and easier. Furthermore, the particles might be able to expand upon incorporation of a protein due to the flexibility of SMA that is less restricted to a specific arrangement than MSP.

### Design of SMA derivatives for specific applications

As discussed above, the special properties of SMA could cause potential problems for specific applications. However, these problems may be solved by designing new types of SMA with modified functional groups may solve these problems. Generally, the structure of SMA should provide plenty of opportunities for chemical modification to tune its properties in order to avoid undesired interactions. Also, the incorporation of fluorescent or radioactive labels or affinity tags is possible and thus an extensive toolbox of different SMAs and their derivatives may be generated for various applications.

## Conclusions

In conclusion, SMA copolymers have been successfully employed in the solubilization of a diverse set of MPs generally showing the superiority of the obtained native nanodisc environment over detergent micelles. The nanodiscs have been subject to complementary studies proving their suitability for a plethora of biophysical and biochemical techniques that allowed insights into protein structure and function as well as native interactions of MPs with both lipids and other proteins. In addition, many other potential applications of the use of SMA copolymers and chemically modified or tailor-made variants thereof remain to be explored and discovered. A major future challenge will be the elucidation of the details of the mode of action of SMA-mediated membrane solubilization, as this will be invaluable for adaptations of the method to more difficult systems or conditions. Altogether, it can be concluded that SMA copolymers are highly promising versatile tools for the study of diverse membrane-related processes and that they are likely to make a large contribution to the field of membrane research in the future.

## Abbreviations

M_n_: Number-average molecular weight
M_w_: Weight-average molecular weight
DLS: Dynamic light scattering
DSC: Differential scanning calorimetry
DMPC: 1,2-Dimyristoyl-*sn*-glycero-3-phosphocholine
EM: Electron microscopy
EPR: Electron paramagnetic resonance
MAnh: Maleic anhydride
MP: Integral membrane protein
MSP: Membrane scaffold protein
NMR: Nuclear magnetic resonance
SMA: Styrene–maleic acid
SMALP: SMA–lipid particle
SMAnh: Styrene–maleic anhydride
TM: Transmembrane
